# Ivabradine for Incessant Atrial Tachycardia in a 1-Year-Old Child After Tetralogy of Fallot Repair

**DOI:** 10.1155/cric/2601345

**Published:** 2025-02-10

**Authors:** Haikal Balweel, Rubiana Sukardi, Eva Miranda Marwali, Rifqi Rizkani Eri, Novaro Adeneur Tafriend, Agus Harsoyo

**Affiliations:** ^1^Department of Cardiology, Gatot Soebroto Army Central Hospital, Jakarta, Indonesia; ^2^Department of Cardiology, Jakarta Heart Center, Jakarta, Indonesia; ^3^Department of Pediatric Cardiology, Jakarta Heart Center, Jakarta, Indonesia; ^4^Department of Pediatric Intensive Care, Jakarta Heart Center, Jakarta, Indonesia

**Keywords:** atrial tachycardia, ivabradine, tetralogy of Fallot, tetralogy of Fallot repair

## Abstract

**Background**: Ivabradine is a novel drug with the ability to reduce heart rate without compromising myocardial contractility or blood pressure. Studies on this drug exist mainly for heart failure and coronary artery disease; for arrhythmia, the studies are limited to small sample trials and case series, mostly unrandomized. Additionally, its evidence for arrhythmia in pediatric patients is limited. Here, we present a case of successful ivabradine administration for incessant atrial tachycardia in a 1-year-old child following tetralogy of Fallot (TOF) repair.

**Case**: A 1-year-old child experienced incessant tachyarrhythmia episodes after TOF repair. Multiple standard therapies, including adenosine, amiodarone, multiple cardioversions, beta-blockers, and digoxin, failed to convert the rhythm. Considering the limited therapy options and existing case reports, ivabradine was administered at a dose of 0.1 mg/kg. Subsequently, sinus rhythm was restored 8 h after its administration, with the heart rate significantly decreased, and the patient returned to a stable hemodynamic status.

**Discussion**: Ivabradine may be an option for incessant atrial tachycardia in pediatric patients. Although most tachyarrhythmias following TOF repair occur due to a reentry mechanism, focal mechanisms can also occur, suggested by timing, gradual acceleration after cardioversion and its response to ivabradine. Future studies are needed to further understand the safety and efficacy of ivabradine for atrial tachycardia in pediatric patients.

## 1. Introduction

Ivabradine is an antagonist of the hyperpolarization-activated cyclic nucleotide-gated (HCN) channel, which controls the funny current (*I*_f_), an ion channel primarily responsible for the activity of cardiac pacemaker cells located in the sinoatrial (SA) node [1]. Blocking of this channel prolongs diastolic depolarization, thereby slowing the SA node firing and resulting in a lowered heart rate. Ivabradine's novelty lies in its ability to reduce heart rate without compromising myocardial contractility or blood pressure, making it a promising treatment option for patients with heart failure, ischemic heart disease, or inappropriate sinus tachycardia who are intolerant to high doses of beta-blockers or calcium channel blockers [1, 2].

However, its efficacy in treating tachyarrhythmias, particularly atrial tachycardia (AT), has not been extensively studied. Initial research on ivabradine primarily focused on patients with heart failure and coronary artery disease, as demonstrated in the BEAUTIFUL study [3], SIGNIFY trial [4], and SHIFT study [5]. Notably, no randomized trials have specifically evaluated ivabradine in tachyarrhythmia patients [3–5].

Arrhythmia is a known complication following the tetralogy of Fallot (TOF) repair. In many cases, arrhythmia in repaired TOF patients arise from macroreentrant circuits due to the repair technique itself, which may involve a ventricular septal defect (VSD) patch, ventriculostomy scar, atriotomy scar, or due to ventricular remodeling secondary to pressure or volume overload [1]. In a multicenter study of adult patients with repaired TOF, 43% had arrhythmias, of which 20% were atrial, including atrial reentrant tachycardia and cavo tricuspid isthmus-dependent atrial flutter (AFL). Additionally, 38% of repaired TOF patients were found to have multiple types of arrhythmias. The occurrence of atrial arrhythmias in TOF is associated with poorer cardiovascular outcomes, thus requires a prompt and optimal treatment [1, 6].

**Figure 1:**
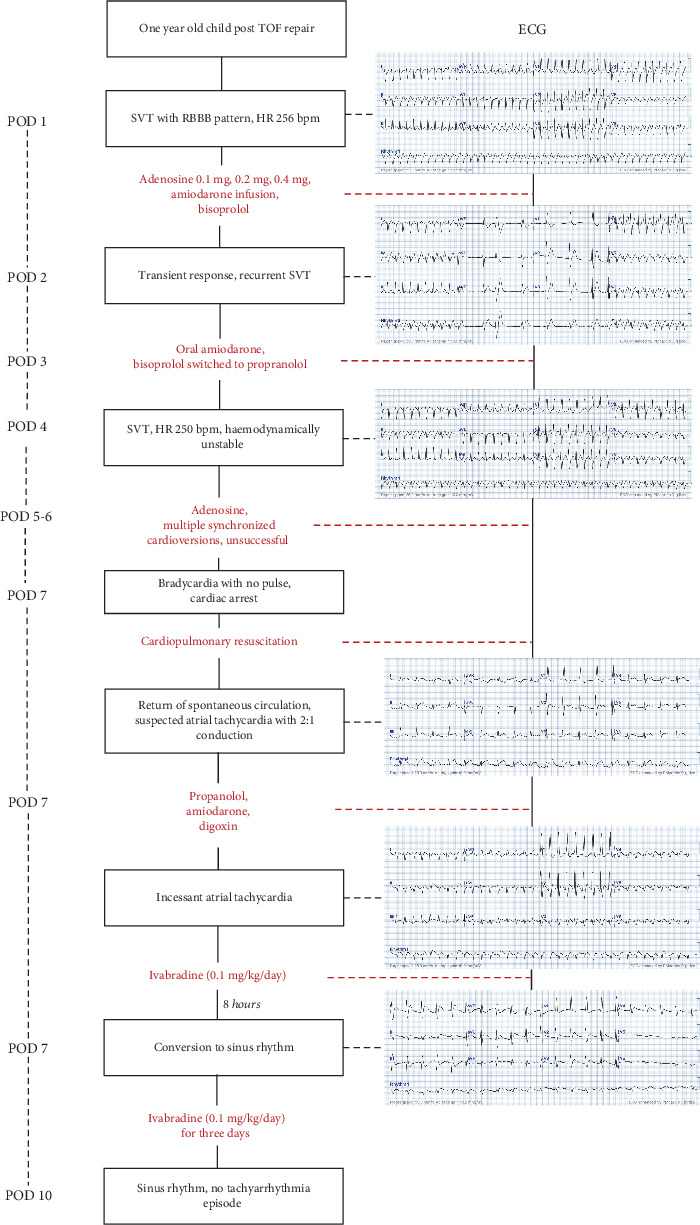
Timeline of tachyarrhythmia episodes with ECG findings, treatments, and clinical responses.

Here, we present a case of incessant AT in a 1-year-old child, occurring 1 day after TOF repair, responsive to ivabradine following refractory responses to standard therapies (Figure 1).

## 2. Case Report

A 1-year-old child underwent TOF repair due to recurrent hypoxic spells. The surgery was considered successful, with postoperative echocardiography showing balanced four chambers, minimal residual VSD, mild residual pulmonary stenosis, and a normal left ventricular (LV) ejection fraction. However, there was one notable finding: right ventricular (RV) dysfunction, evidenced by reduced RV contractility and a tricuspid annular plane systolic excursion (TAPSE) of 4–5 mm.

**Figure 2:**
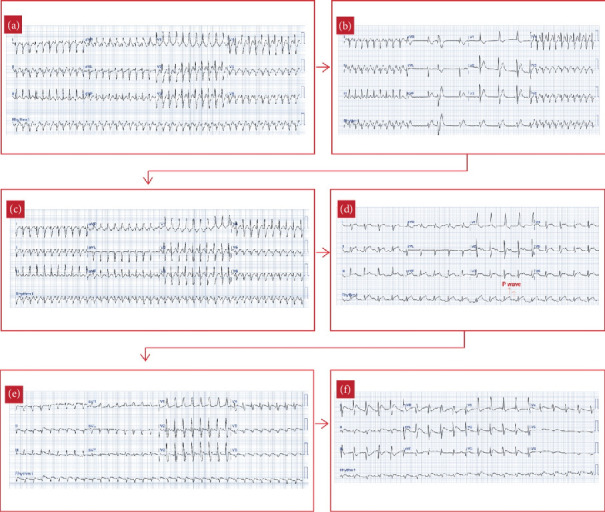
(a) SVT with RBBB pattern on Postoperative Day 1. (b) After amiodarone and bisoprolol administration which did not give good response, followed by stepwise administration of adenosine at doses of 0.1, 0.2, and 0.4 mg, transient responses were seen, but the rhythm quickly reverted to SVT. (c) SVT on Postoperative Day 4 while the patient was put on amiodarone and propranolol, haemodynamically unstable. (d) Following multiple adenosine administration and synchronized cardioversion attempts at 10, 15, and 20 J, in which the patient experienced cardiac arrest and ROSC. P waves were noted at a rate of 300 bpm with a ventricular rate of 150 bpm, indicating atrial tachycardia (AT) as the underlying arrhythmia. (e) Incessant atrial tachycardia despite propranolol, amiodarone, and digoxin administration. (f) Eight hours after the first ivabradine dose (0.1 mg/kg/day, divided into two doses), sinus rhythm was restored.

One day postsurgery, the patient experienced a tachyarrhythmia episode. Electrocardiography (ECG) revealed regular, wide QRS complexes with a right bundle branch block (RBBB) pattern and a heart rate of 259 bpm, suggesting supraventricular tachycardia (SVT) with aberrancy (Figure 2(a)). Immediate treatment was initiated with amiodarone and bisoprolol. Despite these interventions, SVT persisted, leading to stepwise doses of adenosine at 0.1, 0.2, and 0.4 mg. While there was a transient response, the tachyarrhythmia continued to recur with gradual acceleration and was initiated by prolonged PR interval (Figure 2(b)), with the heart rate remaining above 220 bpm, suggesting an atrioventricular nodal reentry tachycardia (AVNRT). On Postoperative Day 3, bisoprolol was switched to propranolol, while amiodarone was maintained. However, by Postoperative Day 4, SVT persisted. Over Postoperative Days 4–7, multiple doses of adenosine and synchronized cardioversions were administered, but SVT kept on recurring with gradual acceleration. At this point, given the incessant nature of the tachyarrhythmia, we began to suspect abnormal automaticity as the underlying mechanism, with junctional ectopic tachycardia (JET) being the most likely.

On Postoperative Day 7, the patient's tachyarrhythmia worsened, with a heart rate of 250 bpm (Figure 2(c)), leading to hemodynamic instability: blood pressure dropped to 64/46 mmHg, and the patient became pale and lethargic. Synchronized cardioversion was attempted three times at 10, 15, and 20 joules, but these efforts were unsuccessful, and the patient went into bradyarrhythmia with no pulse and subsequent cardiac arrest. Fortunately, timely cardiopulmonary resuscitation restored spontaneous circulation. The cardiac rhythm converted to AT, with ECG showing P waves at a rate of around 300 bpm and a ventricular rate of 150 bpm, suggesting AT with a 2:1 atrioventricular (AV) conduction ratio (Figure 2(d)). Given the stable hemodynamic status in this rhythm, we continued propranolol and amiodarone and added digoxin at 8 mcg/kg, yet the AT persisted (Figure 2(e)).

**Figure 3:**
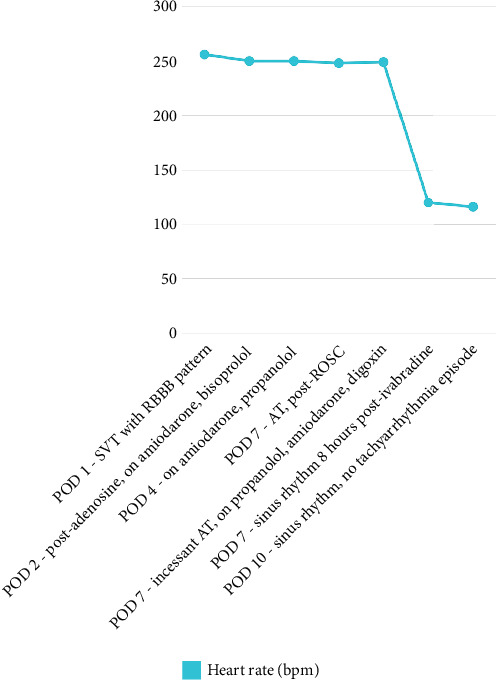
Heart rate at each tachyarrhythmia episode.

The recurrent, gradual reacceleration following termination, along with the persistent nature of the AT, suggested an underlying mechanism of abnormal automaticity, rather than reentry, which guided the decision to use ivabradine. After careful consideration, ultimately on past studies and several case reports, ivabradine was administered at a dose of 0.1 mg/kg, divided into two doses, as an adjunct therapy to the other standard treatments. Eight hours after its first administration, the heart rate remarkably decreased from 210 to 120 bpm, and the ECG showed conversion to sinus rhythm (Figures 2(f) and 3). The patient remained in sinus rhythm, and the hemodynamic status progressively improved. Ivabradine was administered twice daily for 3 days, tapered to once daily on the fourth day, and discontinued on the fifth day when the heart rate stabilized around 80 bpm, successfully converting the AT to sinus rhythm after resistance to standard therapies. The patient required an extended hospitalization due to atelectasis; however, no further tachyarrhythmia occurred.

## 3. Discussion

AT is a relatively rare cardiac arrhythmia that occurs after TOF repair, and ivabradine is also a potential novel therapy for AT but with limited existing evidence. While existing studies describe ivabradine as an antagonist of the HCN channel, primarily located in the SA node, its effectiveness in treating AT in several cases, including this case, suggests that it may act beyond the SA node [2, 6]. In post-TOF repair patients, AT, though less common than JET—the most frequent arrhythmia after TOF repair surgery—can arise from local trauma in a nonsinus atrial tissue. This trauma can induce abnormal automaticity or trigger fibrosis-related intercellular decoupling [7], while sutures, prosthetic materials, and patches may create a nonexcitable tissue substrate, forming a block zone for reentry circuits [7, 8].

In this case, several factors pointed to abnormal automaticity rather than a reentry mechanism. The immediate onset of AT after surgery suggested a focal mechanism, as reentry substrates usually take longer to develop [8]. Focal arrhythmia tends to occur early after surgery. JET can occur within 48 h following the surgery, while focal AT typically emerges 8 to 14 days after the surgery. The mechanism is thought to be related to surgical trauma; however, if the arrhythmia occurs later, it may also involve inflammation and anatomic substrate changes, though these mechanisms remain poorly understood [8]. In contrast, reentrant arrhythmias after heart surgery generally take longer to develop, with incidence increasing over time. This is attributed to the formation of a reentry substrate, which is associated with pressure and volume overload, as well as the gradual process of electrical remodeling [8]. The incessant nature of the tachycardia, being refractory to multiple cardioversions, various antiarrhythmic drugs, beta-blockers, and AV nodal blocking agents, further supports a focal origin, as reentrant arrhythmias are typically interrupted by cardioversion and are less likely to be incessant [9]. Younis et al. added that compared to reentry AT, focal AT tends to be more resistant to beta-blockers, calcium channel-blockers, and Class IC and III antiarrhythmic drugs [2]. Additionally, after multiple cardioversions, the arrhythmia tended to subside briefly, then gradually reappear, showing a “warm-up” phenomenon commonly associated with focal arrhythmias [10]. Finally, the favorable response to ivabradine further suggests abnormal automaticity as the underlying mechanism rather than reentry. Ivabradine creates a negative chronotropic effect by inhibiting the funny current, which is a mixed inward current of sodium and potassium, and is essential for spontaneous depolarization which contributes to abnormal automaticity [2].

Current guidelines recommend adenosine and amiodarone as the initial treatments for wide complex tachycardia in pediatric patients with stable hemodynamics, while synchronized DC cardioversion is advised for unstable patients [11, 12]. In this case, despite administering standard therapies, the AT persisted, even led to haemodynamic instability and cardiac arrest, prompting the use of ivabradine with not many treatment options left. Although this decision may seem unconventional due to limited research on ivabradine's efficacy in pediatric AT, current existing evidence is promising. A prospective study by Banavalikar et al. [6] found that ivabradine was effective in treating 64% pediatric patients with incessant focal AT, though without structural heart disease. Similarly, Xu et al. [13] reported ivabradine's efficacy and safety in a single-center study with pediatric focal AT, showing no recurrence over a 5-month follow-up, though also in patients without structural heart disease [13]. Studies and case reports on ivabradine in AT associated with structural heart disease remain rare, as most arrhythmias in this population are reentry-based, whereas ivabradine primarily targets focal mechanism.

Few reports exist on ivabradine's application in structural heart disease. Janson et al. [14] reported a 14-year-old patient with congenital noncompaction cardiomyopathy and recurrent ectopic AT, initially responding to ivabradine but discontinued after 2 months due to prolonged episodes. Similarly, Hacket, Lin, and Imundo [15] reported a successful ivabradine administration for recurrent ectopic AT in a newborn with hypoplastic left heart syndrome after the Norwood procedure.

Ivabradine is more commonly used to treat postsurgical JET in pediatric patients, although the evidence is also limited to case series. Fernandez et al. [16] proposed an algorithm in which ivabradine could be used after amiodarone for managing postsurgical JET in pediatric patients, based on a case series involving three patients—following aortic valve valvuloplasty, complete heart repair, and complete AV septal defect repair—who were successfully treated with ivabradine. In the proposed algorithm, ivabradine is recommended for patients with a heart rate of > 170 bpm and with a haemodynamic instability refractory to amiodarone, with a dose of 0.1 mg/kg every 12 h, maintained for 72 h [14]. Another case series by Asfour et al. [17] showed successful treatment of congenital JET in two premature neonates using ivabradine [17].

This case report has several limitations. First, while the mechanism of the AT is highly suspected to be due to abnormal automaticity—given its incessant nature, timing postsurgery, and positive response to ivabradine—it cannot be definitively determined without an electrophysiology study. Second, as ivabradine was used as an adjunct to standard therapies rather than as monotherapy, there is potential bias regarding its exact impact on the outcome. However, considering the marked response 8 h after ivabradine administration, with a significant heart rate reduction and stabilization of hemodynamic status, we believe ivabradine was a key factor in this case. This report highlights the potential of ivabradine as a treatment option for focal AT in pediatric patients, though further studies are needed to confirm its efficacy and safety profile in this population.

## 4. Conclusion

Ivabradine can be considered a promising option for treating incessant AT that is unresponsive to standard therapies, particularly in pediatric patients with post-TOF repair. This case suggests that AT occurring shortly after TOF surgery may arise from a focal mechanism linked to trauma to nonsinus atrial tissue, instead of reentry mechanism which tend to occur later due to fibrosis and remodelling. Further studies are required to better understand the efficacy and safety of ivabradine in pediatric patients, as well as to better understand the mechanism of incessant AT in post-TOF repair population.

## Data Availability

The authors have nothing to report.
